# Evaluation of the hematological and clinical biochemical markers of stress in broiler chickens

**DOI:** 10.14202/vetworld.2020.2294-2300

**Published:** 2020-10-31

**Authors:** Chioma U. Nwaigwe, John I. Ihedioha, Shodeinde V. Shoyinka, Chukwuemeka O. Nwaigwe

**Affiliations:** 1 Department of Veterinary Physiology and Pharmacology, Faculty of Veterinary Medicine, University of Nigeria, Nsukka, Nigeria; 2 Department of Veterinary Pathology and Microbiology, Faculty of Veterinary Medicine, University of Nigeria, Nsukka, Nigeria; 3 Department of Animal Health and Production, Faculty of Veterinary Medicine, University of Nigeria, Nsukka, Nigeria

**Keywords:** broiler chickens, hematology, physiological stress, serum biochemistry

## Abstract

**Background and Aim:**

Stress significantly affects health and productivity of animals. There is need for affordable and easy-to-assay markers of physiological stress in broilers. This study evaluated hematological and serum biochemical markers of physiologic stress in broiler chickens.

**Materials and Methods:**

Thirty day-old broiler chickens were assigned to three groups of ten broilers each during the 56-day study. Group 1 broilers served as the control and were not exposed to any stressors; Group 2 broilers were stocked at the stress density of 50 kg BW m^–2^ from day 49 to 56 while Group 3 broilers had their feed withdrawn 36 h before blood collection on day 56. Blood samples were collected on day 49 (pre-stress sample) and day 56 (post-stress sample) from all the birds and subjected to hematological and serum biochemical analysis.

**Results:**

The parameters did not vary significantly (p>0.05) pre-stress. Post-stress, there were significant variations in the heterophil, lymphocyte, and eosinophil counts and in the heterophil to lymphocyte ratio across the three groups. The concentrations of the plasma fibrinogen and serum albumins, sodium, and sodium to potassium ratio of the broilers varied significantly across the groups.

**Conclusion:**

Heterophil, eosinophil, and lymphocyte counts, the heterophil to lymphocyte ratio, plasma fibrinogen, serum albumin, sodium, and sodium to potassium ratio are significant markers of physiological stress in broilers.

## Introduction

Stress can be described as responses elicited by an animal in a bid to adapt or protect itself from the adverse effects of stressors [1]. Physiological stress in animals can be caused by extreme temperature (heat or cold), overcrowding or high stocking density, starvation (feed and/or water deprivation or withdrawal), and harmful handling such as restraint, noise, and transportation. The exposure of birds to stressful conditions can lead to immunological or metabolic consequences. Regression of immune-protective organs (such as the thymus, bursa, and spleen**)**, downsizing of mitochondrial metabolic oxidative capacity, upregulation of avian uncoupling proteins, depletion of antioxidant reserves, and alteration in the pattern of antioxidant enzyme activities have been reported in chickens after experimental stress induction [2,3]. The most common symptoms of stress in broilers include weight loss, increased ­susceptibility to diseases, increased feed conversion ratio, infertility or subfertility, decreased livability, and immune suppression. Constant and prolonged exposure to stress can result in reduced productivity and death [4].

The need for affordable and easy-to-assay markers of physiological stress in broilers is imperative for optimum efficiency of poultry production. In the assessment of stress responses, multiple markers have been proposed. Corticosterone assays are labor-intensive, costly, and require specialized expertise in their analysis; while other techniques of assessing stress responses, such as hematological and some serum biochemical markers, are easier, cheaper, require less expertise/equipment, and are less time-consuming. There is a need for more information on the value and use of these low-cost simple to evaluate markers in poultry.

This study evaluated the hematological and clinical biochemical markers of stress in broiler chickens exposed to two different physiological stressors – food deprivation and high stocking density.

## Materials and Methods

### Ethical approval

The birds were handled humanely all through the study, and the experimental design and protocol for the use of the birds for research were approved by the Institutional Animal Care and Use Committee of the Faculty of Veterinary Medicine, University of Nigeria Nsukka (Approval No. FVM-UNN-IACUC-2019-0921).

### Study location and period

The research was carried out at the Faculty of Veterinary Medicine, University of Nigeria, Nsukka, Enugu state, Nigeria. Nsukka is located at an altitude of 400 m above sea level, on latitude 6° 51ˈE and longitude 7° 29ˈ N. The study was conducted between the months of December 2019 and January 2020.

### Experimental animals and diets

Thirty male broiler chickens (CHI) procured from CHI Company Ibadan, Nigeria, were used for the study. They were kept in deep litter with drinkers and standard feeding troughs. A basal diet was formulated according to the recommendations for starter (1-28 days) and finisher (29-56 days) periods (Table-1) [5].

**Table 1:** Ingredients and nutrients composition of starter and finisher diets.

Ingredient (%)	Starter	Finisher
Maize	55.00	60.00
Soya bean meal	28.00	24.00
Fish meal	3.00	2.00
Palm kernel cake	10.30	15.30
Bone meal	3.00	3.00
Salt (NaCl)	0.25	0.25
Lysine	0.10	0.10
Methionine	0.10	0.10
*Premix	0.25	0.25
Total	100.00	100.00

**Analyzed value (%)**		

Crude protein	21.41	19.85
Metabolizable energy (MJ/kg)	11.85	12.05
Ether extract	3.62	5.40
Crude fiber	4.38	5.60
Ash	6.54	6.30
Calcium	1.11	1.09
Phosphorus	0.86	0.80
*Lysine	1.12	1.02
*Methionine	0.55	0.45

Premix for Starter diet (per kg diet): Vitamin A 15,000 I.U, Vitamin D_3_ 13,000 I.U, thiamin 2 mg, Riboflavin 6 mg, pyridoxine 4 mg, Niacin 40 mg, cobalamine 0.05 g, Biotin 0.08 mg, choline chloride 0.05 g, Manganese 0.096 g, Zinc 0.06 g, Iron 0.024 g, Copper 0.006 g, Iodine 0.014 g, Selenium 0.24 mg, Cobalt 0.024 mg and antioxidant 0.125 g. Premix for Finisher diet (per kg diet): Vitamin A 10,000 I.U., Vitamin D_3_ 12,000 I.U, Vitamin E 20 I.U., Vitamin K 2.5 mg, thiamine 2.0 mg, Riboflavin 3.0 mg, pyridoxine 4.0 mg, Niacin 20 mg, cobalamin 0.05 mg, Pantothenic acid 5.0 mg, Folic acid 0.5 mg, Biotin 0.08 mg, choline chloride 0.2 mg, Manganese 0.006 g, Zinc 0.03 g, Copper 0.006 g, Iodine 0.0014 g, Selenium 0.24 g, cobalt 0.25 g, and antioxidant 0.125 g.

### Experimental design

The birds were brooded together from days 1 to 14. At the end of the 14^th^ day, they were randomly assigned to three groups (Groups 1, 2, and 3) of ten birds each. Birds in Group 1 served as the control and were housed at a standard stocking density of 30 kgBW/m^2^ [6] and were not subjected to feed withdrawal at any point. The birds were kept at an ambient temperature of 24±2°C and given water and feed *ad libitum*. Groups 2 and 3 were maintained at the same temperature conditions as Group 1, but Group 2 birds were stocked at a density of 50 kg BW m^–2^ from days 49 to 56, while feeding was restricted in Group 3 birds for 36 h before blood collection on day 56.

The blood was collected from birds in each group on day 49 and day 56 of the study as the pre-stress and post-stress samples, respectively. All the hematological and serum biochemical determinations were done immediately on sample collection, following standard procedures.

### Hematological determinations

The total white blood cell, thrombocyte, and red blood cell (RBC) counts were enumerated following the hemocytometer method, using Natt and Herrick’s blood cell diluting fluid [7]. Differential white blood cell (WBC) counts were done on thin smears according to the Leishman staining technique [8]. Packed cell volume (PCV) was determined using the microhematocrit method [8].

### Clinical chemistry determinations

Serum corticosterone levels were assayed by competitive enzyme-linked immunoassay using a Chicken corticosterone (CORT) ELISA Kit (Abbkine^®^, Abbkine Inc. China), and a DR-508G Microplate ELISA Reader (Diatek^®^, Wuxi Hiwell Diatek Instruments Co. Ltd., China) with the program mode set at Chicken CORT (O.D. – 450 nm). Plasma fibrinogen levels were determined using the modified heat precipitation method [9] and the bromocresol green reaction was used in the determination of serum albumin levels using an Albumin Test Kit (QCA, Spain) [10]. Serum uric acid concentration, sodium, potassium, calcium, and phosphorus levels were determined using a commercial test kit (QCA, Spain) and results of the determination were read using a Semiautomated Blood Biochemistry Analyzer (CHEM5-V3^®^, Erba Diagnostics, Mannheim, Germany).

### Statistical analysis

Data obtained from the three groups of birds were subjected to a one-way analysis of variance. Variant means were separated using the least significant difference method. The results were presented as mean±standard error, and significance was accepted at p<0.05 and p<0.01. SPSS for Windows version 16 software was used for the analysis.

## Results

### Hematology

Pre-stress, the total WBC count of the broiler groups ranged from a mean of 37.28×10^3^ μ l to 38.50×10^3^ μL and did not vary significantly (p>0.05) (Table-2). Post-stress, the mean total WBC of the Group 3 broilers was significantly (p<0.05) higher than that of the Group 1 broilers. The post-stress total WBC of the Group 2 birds did not differ significantly (p>0.05) from that of Group 1 or Group 2 birds. Pre-stress, the mean heterophil counts of the broiler groups did not vary significantly (p>0.05) and ranged from 7.30×10^3^ μL to 11.35×10^3^ μL. The post-stress heterophil counts of the Group 2 broilers were significantly lower (p<0.01) than those of Groups 1 and 3 broilers, while the heterophil counts of the Group 3 broilers were significantly higher (p<0.01) than those of Groups 1 and 2 birds. Lymphocyte counts among the broiler groups ranged from 22.32×10^3^ μL to 26.33×10^3^ μL pre-stress. The post-stress lymphocyte counts of Group 2 broilers were significantly (p<0.01) higher than those of broilers in Groups 1 and 3, while that of broilers in Group 3 were significantly lower (p<0.01) than those of Groups 1 and 2. The pre-stress heterophil to lymphocyte (H/L) ratio ranged from 0.32 to 0.54 and did not vary significantly (p>0.05) between the groups. Post-stress, the H/L ratio of Group 2 broilers was significantly lower (p<0.01) than that of birds in Groups 1 and 3, while that of broilers in Group 3 were significantly higher (p<0.01) than those of broilers in Groups 1 and 2. The pre-stress eosinophil counts of the broiler groups did not vary significantly (p>0.05) and ranged from 1.90×10^3^ μL to 2.75×10^3^ μL. Post-stress, however, the eosinophil counts of Group 2 broilers were significantly lower (p<0.01) than those of Groups 1 and 3, while, post-stress, the eosinophil counts of Group 3 broilers were significantly higher (p<0.01) than those of Groups 1 and 2 broilers. Pre-stress monocyte counts in the broiler groups ranged from 1.71×10^3^ μL to 2.22×10^3^ μL and both the pre-stress and post-stress values did not vary significantly (p>0.05) across the groups. Both the pre- and post-stress basophil counts did not vary significantly across the broiler groups (p>0.05). The basophil counts ranged from 0.00×10^3^ μL to 0.04×10^3^ μL. The mean pre-stress values of the RBC counts of the broiler groups did not vary significantly (p>0.05) and ranged from 2.93×10^6^ μL to 3.03×10^6^ μL (Table-3). Post-stress, the RBC counts of the Group 3 broilers were significantly higher (p<0.05) than those of the Group 2 birds, though there were no significant differences (p>0.05) between the RBC counts of Groups 1 and 2 broilers. Mean pre-stress PCV in the broiler groups ranged from 28.20% to 28.70% and did not vary significantly (p>0.05) across the groups. The mean post-stress PCV of Group 3 birds was significantly higher (p<0.05) than those of Groups 1 and 2. Mean pre-stress thrombocyte counts of the broiler groups ranged from 164.00×10^3^ μL to 178.00×10^3^ μL and did not vary significantly (p>0.05). Post-stress, however, the mean thrombocyte counts of Groups 2 and 3 broilers were significantly (p<0.01) higher than that of Group 1 broilers.

**Table 2:** The total white blood cell, heterophil, lymphocyte, eosinophil, monocyte, and basophil counts of broilers exposed to varied forms of physiological stress.

Parameter	Response	Group 1 (control)	Group 2 (stocking density stress)	Group 3 (feed withdrawal stress)	Level of significance
Total white blood cell count (×10^3^/μL)	Pre-stress	37.28±1.54	38.30±0.86	38.50±0.35	p=0.773
	Post-stress	36.20±1.48^a^	38.30±0.86^ab^	40.30±0.73^b^	p=0.040
Heterophils (×10^3^/μL)	Pre-stress	11.35±1.23	7.30±1.75	9.61±2.34	p=0.329
	Post-stress	9.36±0.69^a^	5.90±0.97^b^	18.02±0.47^c^	p=0.000
Lymphocytes (×10^3^/μL)	Pre-stress	22.32±1.35	26.33±1.38	23.94±1.58	p=0.166
	Post-stress	20.79±1.35^a^	27.74±0.51^b^	15.13±0.44^c^	p=0.000
Heterophil/lymphocyte ratio	Pre-stress	0.54±0.08	0.32±0.09	0.47±0.14	p=0.351
	Post-stress	0.47±0.04^a^	0.22±0.04^b^	1.20±0.04^c^	p=0.000
Eosinophils (×10^3^/μL)	Pre-stress	1.90±0.32	2.36±0.39	2.75±0.50	p=0.380
	Post-stress	2.98±0.30^a^	1.38±0.20^b^	3.40±0.38^a^	p=0.000
Monocytes (×10^3^/μL)	Pre-stress	1.71±0.46	2.22±0.53	2.17±0.63	p=0.776
	Post-stress	2.65±0.36	3.16±0.59	3.35±0.33	p=0.512
Basophils (×10^3^/μL)	Pre-stress	0.00±0.00	0.00±0.00	0.04±0.04	p=0.401
	Post-stress	0.00±0.00	0.38±0.38	0.00±0.00	p=0.381

a,b,c Alphabetical superscripts in a row indicate a significant difference between the means (p<0.05 or p<0.01).

**Table 3:** The red blood cell count, packed cell volume, and thrombocyte counts of broilers exposed to varied forms of physiological stress.

Parameter	Response	Group 1 (control)	Group 2 (stocking density stress)	Group 3 (feed withdrawal stress)	Level of significance
RBC count (×10^6^/μL)	Pre-stress	3.0±0.10	2.93±0.06	3.03±0.07	p=0.133
	Post-stress	3.11±0.12^ab^	3.06±0.06^a^	3.38±0.09^b^	p=0.049
PCV (%)	Pre-stress	28.20±0.61	28.60±0.58	28.70±0.65	p=0.832
	Post-stress	28.80±0.49^a^	28.70±0.47^a^	30.50±0.31^b^	p=0.010
Thrombocytes (×10^3^/μL)	Pre-stress	176.00±10.45	164.00±7.48	178.00±6.11	p=0.439
	Post-stress	180.00±4.47^a^	209.00±3.48^b^	197.00±4.23^b^	p=0.000

a,b Alphabetical superscripts in a row indicate a significant difference between the means (p<0.05 or p<0.01). RBC=Red blood cell count, PCV=Packed cell volume.

### Clinical chemistry

The mean serum corticosterone concentration of the broilers ranged from 7.21 ng/L to 9.46 ng/L, pre-stress, and 19.15 ng/L to 22.52 ng/L post-stress (Table-4). The pre-stress and post-stress values did not vary significantly (p>0.05) across the groups. The mean pre-stress values of plasma fibrinogen did not vary significantly (p>0.05) and ranged from 381.70 mg/dL to 440.34 mg/dL. Post-stress, the plasma fibrinogen levels of Groups 2 and 3 broilers were significantly (p<0.01) higher than that of Group 1 broilers.

**Table 4:** The blood corticosterone, fibrinogen, albumin, and uric acid levels of broilers exposed to varied forms of physiological stress.

Parameter	Response	Group 1 (control)	Group 2 (stocking density stress)	Group 3 (feed withdrawal stress)	Level of significance
Corticosterone (ng/L)	Pre-stress	9.46±1.12	7.15±0.41	7.21±1.25	p=0.196
	Post-stress	19.93±2.11	19.15±1.52	22.52±2.86	p=0.437
Fibrinogen (mg/dL)	Pre-stress	429.71±37.56	381.70±29.77	440.34±27.32	p=0.395
	Post-stress	350.40±17.76^a^	487.94±11.72^b^	494.79±16.24^b^	p=0.000
Uric acid (mg/dL)	Pre-stress	2.76±0.30^a^	3.56±0.28^b^	3.66±0.27^b^	p=0.061
	Post-stress	3.40±0.28^a^	5.44±0.49^b^	3.67±0.20^a^	p=0.001
Albumin (g/dL)	Pre-stress	1.58±0.06	1.69±0.05	1.75±0.06	p=0.154
	Post-stress	1.88±0.05^a^	2.20±0.04^b^	2.11±0.04^b^	p=0.000
Uric acid/albumin ratio	Pre-stress	1. 74±0.18	2.14±0.19	2.11±0.15	p=0.233
	Post-stress	1.80±0.13^a^	2.47±0.20^b^	1.74±0.09^a^	p=0.003

a,b Alphabetical superscripts in a row indicate a significant difference between the means (p<0.05 or p<0.01).

Pre-stress, the mean serum uric acid concentration of the broilers ranged from 2.76 mg/dL to 3.66 mg/dL and did not vary significantly (p>0.05) (Table-4). However, post-stress, Group 2 broilers had a significantly (p<0.01) higher mean serum uric acid concentration (5.44 mg/dL) compared to that of Group 1, while the serum uric acid level of Group 3 broilers did not differ significantly (p>0.05) from that of Group 1. The mean serum albumin concentration of the broilers did not vary significantly (p>0.05) across the groups and ranged from 1.58 g/dL to 1.75 g/dL. The post-stress serum albumin concentrations of Groups 2 and 3 broilers were significantly (p<0.01) higher than that of Group 1 broilers. The pre-stress uric acid to albumin ratio of the broilers ranged from 1.74 to 2.11 and did not vary significantly (p>0.05) across the groups. Post-stress, the broilers from Group 2 had a significantly (p<0.05) higher uric acid to albumin ratio than Groups 1 and 3.

Pre-stress, the mean serum sodium concentration of the broilers ranged from 128.06 mEq/L to 129.26 mEq/L and did not vary significantly (p>0.05) among the groups (Table-5). Post-stress, the mean serum sodium levels of Groups 2 and 3 were significantly (p<0.01) higher than that of Group 1. Pre-stress, the mean serum potassium level of the broilers in Group 3 was significantly (p<0.05) higher than that of Groups 1 and 2. During post-stress sampling, however, broilers in Groups 2 and 3 had significantly (p<0.05) higher levels of serum potassium compared to that of Group 1 broilers. The mean pre-stress serum sodium to potassium ratio of Group 3 broilers was significantly (p<0.05) lower than that of Group 1 but did not differ significantly (p>0.05) from that of Group 2 broilers. However, post-stress both Groups 2 and 3 broilers had a significantly (p<0.01) lower serum sodium to potassium ratio compared to that of Group 1.

**Table 5:** The serum electrolyte levels of broilers exposed to varied forms of physiological stress.

Parameter	Response	Group 1 (control)	Group 2 (stocking density stress)	Group 3 (feed withdrawal stress)	Level of significance
Sodium (mmol/L)	Pre-stress	129.10±1.04	129.26±1.12	128.06±0.94	p=0.676
	Post-stress	140.45±0.67^a^	135.68±1.11^b^	136.21±0.76^b^	p=0.001
Potassium (mmol/L)	Pre-stress	3.65±0.17^a^	3.82±0.19^a^	4.22±0.20^b^	p=0.033
	Post-stress	2.76±0.20^a^	3.90±0.25^b^	4.10±0.20^b^	p=0.014
Sodium/potassium ratio	Pre-stress	36.11±1.83^a^	34.71±1.99^ab^	30.35±1.21^b^	p=0.023
	Post-stress	53.56±4.10^a^	35.98±2.16^b^	33.88±1.47^b^	p=0.002
Calcium (mg/dL)	Pre-stress	8.84±0.19^a^	9.00±0.18^a^	9.75±0.16^b^	p=0.030
	Post-stress	9.88±0.10	10.22±0.13	10.20±0.19	p=0.240
Phosphorus (mg/dL)	Pre-stress	6.08±0.52	6.26±1.10	6.41±1.32	p=0.273
	Post-stress	6.55±0.35	6.43±0.28	6.33±0.26	p=0.594
Calcium/phosphorus Ratio	Pre-stress	1.45±0.44	1.44±0.56	1.52±0.49	p=0.149
	Post-stress	1.51±0.07	1.59±0.06	1.61±0.06	p=0.096

a,b Alphabetical superscripts in a row indicate a significant difference between the means (p<0.05 or p<0.01).

The serum calcium levels of the broilers in Group 3 were significantly (p<0.05) higher than those of Groups 1 and 2, which did not vary significantly (p>0.05) from each other (Table-5). Post-stress, the mean serum calcium levels of the broilers did not vary significantly (p>0.05) across the groups. The mean serum phosphorus levels of the broiler groups ranged from 6.08 mg/dL to 6.41 mg/dL pre-stress and 6.33 mg/dL to 6.55 mg/dL post-stress. Neither the pre-stress nor post-stress values varied significantly (p>0.05) across the groups. Furthermore, the calcium to phosphorus ratios of the broilers ranged from 1.45 to 1.52 pre-stress and 1.51 to 1.61 post-stress across the groups. Neither pre-stress nor post-stress calcium to phosphorus ratios varied significantly (p>0.05).

## Discussion

Both stressors (increase in stocking density of the broiler chickens and withdrawal of their feed) resulted in the elevation of the total WBC count, which was significantly higher in the feed withdrawal stress group. This trend of increase in total WBC counts had been documented in humans in cases of psychological stress and in animal models exposed to social stress [11,12]. Stress induces the nerve fibers to release noradrenaline into the bloodstream, which triggers signals in the bone marrow to increase production of hematopoietic stem cells, especially white blood cells [13].

The heterophil and lymphocyte counts, and consequently the heterophil to lymphocyte ratio, can vary depending on the stressor [14]. There are many consistent reports on increased H/L ratios in avian species under heat stress [15-19]. In our study, this was also observed in the birds subjected to feed withdrawal stress; however, the high stocking density stress group had a low H/L ratio. Reports on the effect of high stocking density on H/L ratio are highly variable, with either an increase at 27.2 kgBW/m^2^ and 45 kgBW/m^2^ or no significant effect at 40 kgBW/m^2^ [20-22]. Differences in specific stocking density, housing, environmental conditions, and management practices are factors that may have influenced these differences [23].

The significantly lower eosinophil levels in the broilers subjected to stocking density stress concur with reports showing that a reduction in circulating eosinophil numbers is an indicator of stress in animals [24,25]. A reduction in the eosinophil numbers in blood can be a distinguishing factor between leukocyte responses due to infection and that due to stress [26]. Exposure of the broilers to the two types of stressors used in our study had no significant effect on monocyte and basophil counts, which implies that changes in these blood cell counts are not markers of physiological stress.

Animals subjected to heat stress have decreased hematocrit values [27,28]. However, in the present study, the group of broilers with restricted feeding had higher mean hematocrit values and erythrocyte counts than that of other groups. Increased PCV may be related to the increased metabolic activity required to meet the increased energy demands for maintenance and growth during stressful conditions [29]. We found that the mean thrombocyte counts were significantly higher in the two broiler groups subjected to stress than that of the group not exposed to any stressors. Increases in thrombocyte count act as markers of mental and physical stress in humans [30,31].

Corticosterone is a commonly and frequently assessed biomarker of stress in birds [32-34]. Increased plasma or serum corticosterone has been recorded in poultry during transportation stress and heat stress [23,35]. However, high stocking density and feed withdrawal did not elicit any significant alteration in the corticosterone level of the broiler chickens when compared to that of the control group. Our findings agree with reports that stocking density stress leads to no significant effect on corticosterone concentrations [21,36,37]. Serum corticosterone has also been reported to be an unreliable measure of chronic stress due to hunger [23,38]. Furthermore, corticosterone is highly mobilized when a bird is captured and handled for long, making its measurement and interpretation difficult [39,40]. The H/L ratio may be a more consistent indicator of social stress than plasma corticosteroid levels [41]. Corticosterone assay kits are expensive and analysis of samples requires the use of sensitive and expensive equipment (microplate reader) and specialized software, while the method for measuring H/L ratios requires only stain, microscope slides, a microscope, and immersion oil [42].

Acute-phase proteins, first described as early reactants to infectious diseases and inflammation, are also valuable biomarkers of stress [43]. In humans, fibrinogen responses increase with psychophysiological and chronic work stress [44,45]. Albumin decreases in serum following chronic stress but can also increase in case of stress-hemoconcentration or dehydration, as recorded in transport stress [46]. Our findings of significantly higher levels of plasma fibrinogen and albumin in the broiler groups exposed to stress concur with these reports.

The serum uric acid concentration was significantly higher post-stress in the high stocking density group than in the other groups. Circulating uric acid is a measure of protein catabolism and its increase reflects increased protein or amino-acid catabolism [47]. In fish [48], high stocking density resulted in increased serum uric acid levels due to increased gluconeogenesis and protein catabolism, which ­corroborates the higher post-stress serum uric acid concentration recorded in the present study for the stocking density stress group.

This study showed that post-stress serum sodium levels were significantly lower in both the high-density stocking group and the feed withdrawal group; this has also been reported in experimentally induced stress in broilers [49]. The serum potassium levels of the broiler chickens exposed to high stocking density and feed withdrawal stress were significantly higher than that of the control group, resulting in the broilers exposed to stress having significantly lower sodium to potassium ratios compared to that of the control group. The inverse relationship between serum sodium and potassium concentrations is likely based on the flux of electrolytes between intra and extracellular spaces [50]. The variations in the pre- and post-stress serum electrolyte concentrations in our study might be attributed to fluctuations in water balance, rate of fluid intake, and frequency of excretion [51].

Increased serum calcium levels have been reported in mice under stress conditions [52]. However, we observed no significant differences in the calcium and phosphorus serum levels or the calcium to phosphorus ratios of broilers exposed to stress compared to that of the control group.

## Conclusion

We found that heterophil, lymphocyte, eosinophil, thrombocyte counts, H/L ratio, serum sodium, and uric acid are significant markers of physiological stress in broilers subjected to stocking density or feed withdrawal stress. Most of the hematological and basic biochemical parameters are easy to assay, inexpensive, and more reliable than corticosterone, which is costly and inconsistent.

## Authors’ Contributions

CUN, JII, SVS, and CON designed the work. CUN, JII, and CON collected the data and did the laboratory work. JII and SVS supervised the work. CUN analyzed the data and drafted the article. All authors read and approved the final manuscript.
